# Impaired cell envelope resulting from *arcA* mutation largely accounts for enhanced sensitivity to hydrogen peroxide in *Shewanella oneidensis*

**DOI:** 10.1038/srep10228

**Published:** 2015-05-15

**Authors:** Fen Wan, Yinting Mao, Yangyang Dong, Lili Ju, Genfu Wu, Haichun Gao

**Affiliations:** 1 Institute of Microbiology and College of Life Sciences, Zhejiang University, Hangzhou, Zhejiang, 310058, China

## Abstract

Oxidative stress is one of the major challenges that *Shewanella* encounter routinely because they thrive in redox-stratified environments prone to reactive oxygen species (ROS) formation, letting alone that ROS can be generated endogenously. As respiration is the predominant process for endogenous ROS, regulators mediating respiration have been demonstrated and/or implicated to play a role in oxidative stress response. In our efforts to unveil the involvement of global regulators for respiration in the oxidative stress response, we found that loss of the Arc system increases *S. oneidensis* sensitivity to H_2_O_2_ whereas neither Fnr nor Crp has a significant role. A comparison of transcriptomic profiles of the wild-type and its isogenic *arcA* mutant revealed that the OxyR regulon is independent of the Arc system. We then provided evidence that the enhanced H_2_O_2_ sensitivity of the *arcA* mutant is due to an increased H_2_O_2_ uptake rate, a result of a cell envelope defect. Although one of three proteases of the ArcA regulon when in excess is partially accountable for the envelope defect, the major contributors remain elusive. Overall, our data indicate that the Arc system influences the bacterial cell envelope biosynthesis, a physiological aspect that has not been associated with the regulator before.

Microorganisms live in environments abundant with various perturbations and therefore develop general and specific stress responses. Oxidative stress, caused by reactive oxygen species (ROS), is arguably the most unavoidable to cells in aerobic environments because cells on their own inevitably and continuously produce ROS from autooxidation of components of the respiratory chain 1. ROS damages a variety of cellular macromolecules, such as DNA, RNA, proteins, and lipids. To keep the concentration of ROS at an acceptable level and to repair oxidative damages, cells evoke a cellular mechanism responding to oxidative stresses that permits survival. In bacteria, the primary defense comprises superoxide dismutase, catalase, and peroxidase that directly remove excess ROS and glutathione/glutaredoxin/thioredoxin that help maintain an intracellular reducing environment thus limiting ROS damages 2. In parallel, bacterial cells are equipped with a repairing system, consisting of endonuclease, proteolytic, and lipolytic enzymes, which functions as the secondary defense by removing damaged cellular components.

In bacteria, the oxidative stress response is predominantly mediated by four transcriptional regulators, OxyR, SoxRS, PerR, and OhrR 1,3). The OxyR system, widely found in Gram-negative bacteria, responds to hydrogen peroxide (H_2_O_2_) mostly as an activator, whereas the less omnipresent SoxRS system is activated by redox-active compounds to prevent damages on macromolecules from superoxides 4. In many Gram-positive bacteria, PerR takes place of OxyR responding to H_2_O_2_ as a repressor. OhrR, conserved among Gram-negative and -positive bacteria, is specific for responding to organic peroxide (OP) 3. In addition, there exists the extensive connectivity between the regulons specific to the oxidative stress response and other regulatory systems. For instance, RpoS (σ^54^) is important for expression of many genes that are induced under a variety of stresses and Fur, the principal regulator of iron homeostasis, influences transcription of some members of the ROS-specific regulons by interacting with their Fur-binding sequences 5. Moreover, two redox-sensing global regulatory systems mediating the transition from aerobic to anaerobic metabolism, Fnr (*f*umarate and *n*itrate reduction *r*egulator) and Arc (*a*erobic *r*espiration *c*ontrol) two-component system, have been implicated to have an important role in the resistance to ROS-induced damage 6,7,8,9,10,11,12.

*Shewanella*, facultative Gram-negative anaerobes renowned for their remarkable anaerobic respiratory abilities, become a research model for investigating redox transformations of a variety of inorganic and organic chemicals, with *S. oneidensis* as most intensively studied 13. Compared to *Escherichia coli*, *S. oneidensis* is hypersensitive to H_2_O_2_ and to all wavelengths of solar UV, UV and ionizing radiation, a process linked to ROS generation 14. Surprisingly, despite intense interest in the mechanisms by which *Shewanella* copes with environmental stresses 15, how this group of bacteria responding to oxidative stresses has not been investigated until recently 16,17. Like most other Gram-negative bacteria, *S. oneidensis* uses OxyR and OhrR as the master regulators to mediate its response to H_2_O_2_ and OPs respectively, but lacks an analogue of SoxR 16,17. The OxyR and OhrR regulons appear to be functionally intertwined as both OxyR and OhrR systems can sense and respond to H_2_O_2_ and OP agents 17. Interestingly, while *S. oneidensis* is fully equipped with a whole package of enzymes to scavenge ROS, the genes encoding proteins for the secondary defense are either missing or unresponsive to the stress 16. Moreover, OxyR plays an important role in phase induction and biofilm formation 18.

*S. oneidensis* differs substantially from *E. coli* in the proteins involved in sensing and maintaining the cellular redox state. The *S. oneidensis* Fnr plays an extremely limited role in regulation of respiration and is certainly not critical in controlling transition from aerobic to anaerobic metabolism 19,20,21. Instead, Crp (*c*yclic-AMP *r*eceptor *p*rotein) has been shown to be the dominant regulator for respiration although this protein is unlikely to be able to sense redox changes directly due to the lack of redox-sensing domains 22,23,24,25. Furthermore, *S. oneidensis* possesses an atypical Arc system in which function of the sensor kinase is fulfilled by two proteins, ArcS and HptA 26,27. Unlike its counterpart in *E. coli*, this atypical system plays an important role in aerobic respiration without interfering with expression of genes encoding components of the tricarboxylic acid (TCA) cycle 28,29,30.

Given that oxygen availability is generally intertwined with oxidative stress, in this study we attempted to determine the involvement of *S. oneidensis* Fnr, Crp, and Arc in oxidative stress response. We showed that loss of the Arc system elevates sensitivity to H_2_O_2_ whereas neither Fnr nor Crp has a significant role in response to the agent. A comparison of the transcriptomic changes elicited by H_2_O_2_ between the wild type and *arcA*-deficient strains demonstrated that the Arc system is not an important player in co-regulating genes belonging to the OxyR regulon. The enhanced H_2_O_2_ sensitivity of the *arcA* mutant was then linked to an increased H_2_O_2_ uptake rate, a result of a cell envelope defect. We then showed that this defect is partially attributable to overproduction of SO1915, one of three proteases of the ArcA regulon. Moreover, it seems that the impaired envelope resulting from the *arcA* mutation is rather complex, unlikely depending on a single gene. The present study for the first time links the Arc system with the biosynthesis of bacterial cell envelope.

## Methods

### Bacterial strains, plasmids, and culture conditions

A list of all bacterial strains and plasmids used in this study is given in Table 1. Information for primers used in this study was available upon request. *E. coli* and *S. oneidensis* strains under aerobic conditions were grown in Lysogeny Broth (LB, Difco, Detroit, MI) medium 31, which was modified to contain tryptone (10 g/L), yeast extract (5 g/L), and NaCl (5 g/L), at 37 °C and 30 °C for genetic manipulation. When needed, the growth medium was supplemented with chemicals at the following concentrations: 2, 6-diaminopimelic acid (DAP), 0.3 mM; ampicillin sodium, 50 μg/ml; kanamycin sulfate, 50 μg/ml; and gentamycin sulfate; 15 μg/ml.

### In-frame mutant construction and complementation

In-frame deletion strains for *S. oneidensis* were constructed using the *att*-based fusion PCR method as described previously 32. In brief, two fragments flanking the target gene were amplified by PCR with the gene specific primers, which were joined by the second round of PCR. The fusion fragments were introduced into plasmid pHGM01 by using Gateway BP clonase II enzyme mix (Invitrogen) according to the manufacturer’s instruction, resulting in mutagenesis vectors in *E. coli* WM3064, which were subsequently transferred into relevant *S. oneidensis* strains via conjugation. Integration of the mutagenesis constructs into the chromosome was selected by resistance to gentamycin and confirmed by PCR. Verified transconjugants were grown in LB broth in the absence of NaCl and plated on LB supplemented with 10% sucrose. Gentamycin-sensitive and sucrose-resistant colonies were screened by PCR for deletion of the target gene. All mutants were verified by sequencing the mutated regions.

For complementation, a DNA fragment containing gene of interest and its native promoter was generated by PCR and introduced into pHG101 33. The resulting complementation vector was maintained in *E. coli* WM3064, verified by sequencing, and transferred into relevant mutation strains via conjugation.

### Physiological characterization of *S. oneidensis* in response to H_2_O_2_ and SDS

Growth of *S. oneidensis* strains generated in this study was measured by recording the optical density at 600 nm (OD_600_) values in triplicate with the wild-type as the control in M1 defined medium containing 0.02% (w/v) of vitamin-free Casamino Acids and 15 mM lactate as described previously 29. Impacts of H_2_O_2_ on *S. oneidensis* strains were assessed by three approaches. First, minimum inhibitory concentrations (MIC) of H_2_O_2_ and SDS were determined in LB as described previously 16. Second, disk diffusion assays were carried out. Briefly, cultures of mid-log phase (~0.4 of OD_600_, same afterwards) were properly diluted and spread onto fresh LB plates (200 μl of culture; approximately 10^6^ colony forming units (cfu). Paper discs of 6 mm in diameter loaded with 10 μl H_2_O_2_ of 5 M were placed onto the bacterial lawn and plates were incubated at 30 ^o^C for 16 h. Third, survival of *S. oneidensis* strains was assayed. H_2_O_2_ was added to the mid-log phase cultures to a final concentration of 1.0 mM and samples were taken at 5 and 30 min. The cultures were serially diluted with fresh LB and plated onto LB plates. Plates from dilutions that gave 100 to 250 cfu were counted.

To measure H_2_O_2_ consumption, mid-log phase cells were collected, washed twice in 50 mM NaHPO_4_ buffer (pH 7.0) and resuspended in the same buffer to an OD_600_ of 0.1. H_2_O_2_ was added to a final concentration of 0.5 mM and the cells were incubated at 30 ^o^C. Aliquots were assayed for remaining H_2_O_2_ 1, 5, and 10 min after the treatment began using the FOX method 34.

The susceptibility of *S. oneidensis* strains to SDS was assessed by a drop plate assay. Cells at mid-log phase were adjusted to approximately 10^8^ CFU/ml with fresh LB medium, followed by 6 10-fold serial dilutions. Five microliters of each dilution was spotted onto LB plates containing SDS. The plates were incubated for 24 h or longer before being read. The cell envelope defect was also assessed by adding SDS into mid-log phase cultures and monitoring the optical density reduction.

### Microarray analysis

Microarray analysis was performed essentially the same as described previously 16. In brief, for each strain under aerobic conditions, 20 ml of LB in a 100 ml shake flask was inoculated with fresh overnight culture to OD600 of 0.01 and shaken on a rotary platform (250 rpm) until the mid-log phase. Cultures were divided into two parts; one was used as the untreated control and the other was applied to H_2_O_2_ at a final concentration of 0.2 mM for a treatment of 5 min. All cultures were centrifuged at 14000 rpm for 30 s at room temperature and the pellets were frozen immediately in liquid nitrogen and stored at –80 °C. In total, four biological replicas under each condition were prepared. DNA microarrays, total RNA extraction, cDNA labeling, hybridization, slide scanning, and data analysis were described previously 29,35). Refer to NCBI GEO accession number GSE31053 for raw microarray data.

### Electrophoretic motility shift assay (EMSA)

Expression and purification of His-tagged *S. oneidensis* ArcA has been described before 29,36). Phosphorylation of purified ArcA was performed in buffer containing 100 mM Tris/HCl (pH 7.0), 10 mM MgCl_2_, 125 mM KCl, 50 mM dilithium carbamoyl phosphate for 60 minutes at room temperature. The probes used for EMSA were prepared by PCR with ^33^P end-labeled primers 29. The binding reaction was performed with ~25–50 fmol (~2-5 nM) labeled probes and various amounts of protein in 12 μl binding buffer containing 100 mM Tris/HCl (pH 7.4), 20 mM KCl, 10 mM MgCl_2_, 2 mM DTT, 0.2 μg/μl poly(dI·dC), and 10% glycerol at 15 °C for 60 minutes and resolved on pre-run 4.8% polyacrylamide native gels. Band shifts were visualized by autoradiography.

### Expression analysis

β-Galactosidase activity assay was performed to determine gene expression. DNA fragments of ~400 bp covering sequences upstream of target genes were amplified and placed in front of the full-length *E. coli lacZ* gene on plasmid pHGEI01 37. The resulting vector was transformed into *E. coli* WM3064, verified by sequencing and then transferred into *S. oneidensis*. Cells of mid-log phase prepared as for the microarray analysis were harvested by centrifugation, washed with phosphate-buffered saline (PBS, pH 7.0), and lyzed with the lysis buffer (0.25 M Tris/HCl, pH 7.5, 0.5% Triton X-100). The resulting soluble protein was collected after centrifugation and used for enzyme assay by adding the aliquot of the o-nitrophenyl-β-D-galactopyranoside (ONPG) (4 mg/ml). The protein concentration of the cell lysates was determined using a Bradford assay with BSA as a standard (Bio-Rad). β-galactosidase activity were determined by monitoring color development at 420 nm using a Synergy 2 Pro200 Multi-Detection Microplate Reader (Tecan), presented as Miller units.

For catalase activity analysis, proteins in the cell lysates prepared as described above were separated using 10% non–denaturing PAGE. Catalase was detected by the activity staining method 38

### *In vivo* diffusion of H_2_O_2_

Five ml mid-log cells were pelletted by centrifugation for 5 min at 4500 rpm, suspended in 1 ml of 50 mM sodium phosphate buffer, pH 7.2. Aliquots of the suspension in a 200 μl volume were incubated with 0.5 mM H_2_O_2_ for 5 min and then vacuum-filtered using polycarbonate filters of 0.025 μm (Millipore). The flow-through, taken as extracellular portion, was collected and cells on the filter were suspended with 2 ml of the same suspension buffer and disrupted by French press, which was taken as intracellular portion. Both the extracellular and intracellular fractions were incubated separately with 2 μM horseradish peroxidase and 2 μM scopoletin and the resultant fluorescence was measured at 350 nm (excitation) and 460 nm (emission) using a Synergy 2 Pro200 Multi-Detection Microplate Reader (Tecan) as described somewhere else 39. The background fluorescence from a control cell suspension not exposed to H_2_O_2_ was subtracted and resulting values were normalized by protein concentrations. H_2_O_2_ uptake was determined as the extracellular/intracellular fluorescence ratio.

### Other analyses

Protein subcellular localization prediction was carried out with PSORTb 40. Genome screening for ArcA–binding sites based on established weight matrixes from *S. oneidensis* was performed using regulatory sequence analysis tools (RSAT) 41. Experimental values were subjected to statistical analyses and presented as means ± SD (standard deviation). Student’s *t*-test was performed for pairwise comparisons of groups.

## Results

### *S. oneidensis arcA* mutant shows an enhanced sensitivity to H_2_O_2_

**Figure 1:**
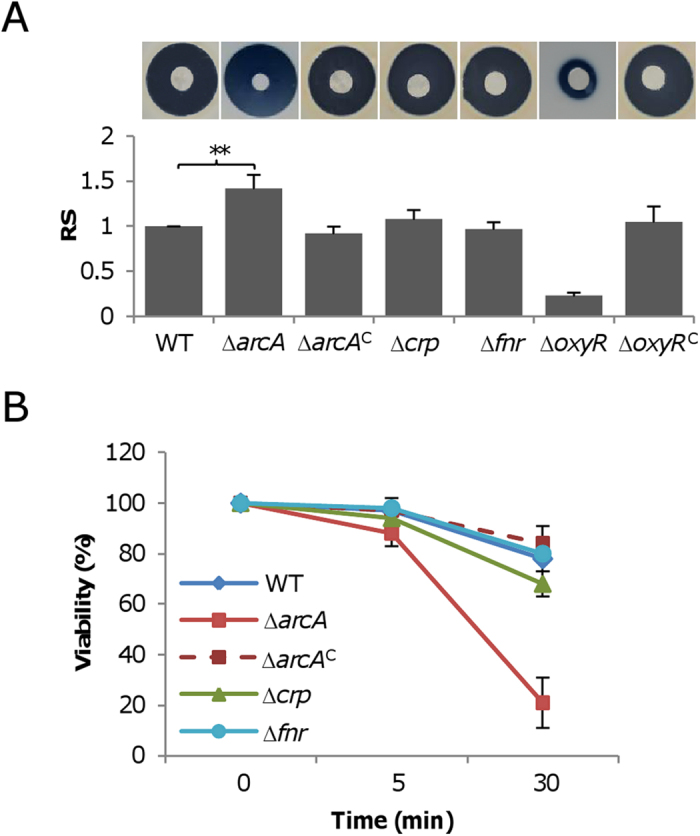
Loss of ArcA increases the H_2_O_2_ sensitivity of *S. oneidensis.* ∆*oxyR* was included for comparison, which had reduced sensitivity because derepression of the predominant catalase KatB. ∆*arcA*^C^ and ∆*oxyR*^C^ represents these mutants carrying a copy of the corresponding missing gene for complementation. (**A**) Effects of *arcA*, *crp*, and *fnr* mutations on H_2_O_2_ inhibition. Cultures of mid-log phase were properly diluted and plated onto LB plates. Paper discs of 6 mm in diameter loaded with 10 μl 5 M H_2_O_2_ were placed onto the bacterial lawn grown for 6 h. Results shown are from 18 h after the discs were in place. For data presentation, the relative sensitivity (RS) (*y axis)* of each mutant strain was calculated by normalizing its average diameter (*n* = 4) to the averaged diameter (n = 6) of the wild-type strain. Error bars represent SD. Asterisks indicate statistically significant difference (*P < 0.05; **P < 0.01; ***P < 0.001). (**B**) Survival assay. H_2_O_2_ was added to mid-log-phase cultures to the final concentration of 1 mM. After 5 min and 30 min, samples were properly diluted and plated onto LB plates. Colony counting was done after 24 h. The data reported represent the means (*n* = 3) + SD.

As a starting point for this work, we examined physiological impact of H_2_O_2_ on the *S. oneidensis arcA*, *crp*, and *fnr* in-frame deletion strains. The MIC values for the wild-type, ∆*arcA*, ∆*crp*, and ∆*fnr* strains were 1.25, 0.625, 1.25, and 1.25 mM at 30 ^o^C, respectively. Because of differences in growth rates 20, incubation times for tested strains were adjusted accordingly, relative to 16 h of the wild-type. By using a disk diffusion test, susceptibility of these *S. oneidensis* strains to H_2_O_2_ was measured (Fig. 1A). Instead of the low density of cells used in determining MIC, H_2_O_2_ discs were applied to a bacterial cell lawn. It was found that the *fnr* deletion strain was indistinguishable from the wild type. In contrast, the Δ*arcA* strain was significantly more sensitive to H_2_O_2_, with a clear zone approximately 1.4-fold greater than that of the wild-type. In the case of the *crp* gene, it appeared that its loss resulted in a slightly increase in susceptibility. In addition, the viability of mid-log phase cells in the presence of 1 mM of H_2_O_2_ was estimated by counting cfu at 5 and 30 min postexposure (Fig. 1B). Similar to results from the disk diffusion test, the treatment did not reveal any significant difference in viability between the wild-type and Δ*fnr* strains. Loss of either the *crp* or *arcA* gene affected the survival when compared to the wild-type. Apparently, the Δ*arcA* strain was substantially more vulnerable to H_2_O_2_ than the wild-type whereas the Δ*crp* strain was impaired marginally. Given that Crp is a global regulator involved in many metabolic processes under both aerobic and anaerobic conditions 22,23,24,25, we speculate that the slightly increased susceptibility and reduced viability upon the H_2_O_2_ treatment are likely due to its pleiotropic effect on the physiology. On the contrary, reasons underlying increased sensitivity of *S. oneidensis* to H_2_O_2_ resulting from the loss of the Arc system deserve further investigation, as this system has been implicated in the oxidative stress in many bacteria but the proposed mechanisms are diverse 6,9,10,42).

### OxyR functions normally in the Δ*arcA* strain

**Figure 2:**
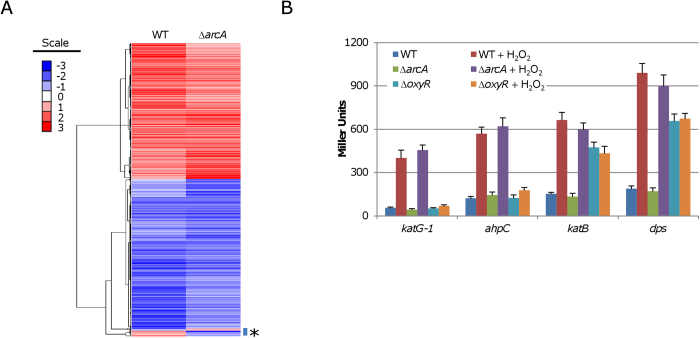
OxyR regulo*n* is independent of the Arc system. (**A**) Clustered heat map of transcriptomes of the wild-type and ∆*arcA* mutant strains in response to H_2_O_2_. Cultures of mid-log phase were divided into two parts; one was collected as the untreated and the other was treated with 0.2 mM H_2_O_2_ and collected 5 min after as the treated. For each strain, the treated/untreated ratios were used for clustering and shown in color scale. Asterisk indicates genes that were significant different between two strains. (**B**) Expression of signature members of the OxyR regulon in the wild-type and ∆*arcA* mutant strains in response to H_2_O_2_. The data reported represent the means (*n* *= *3) ± SD.

To screen for candidate genes for the observed phenotype caused by the loss of ArcA, a microarray analysis was conducted to illustrate the transcriptomic differences elicited by the *arcA* mutation. We sampled mid-log-phase cells 5 min after the addition of 0.2 mM H_2_O_2_ in order to examine the transcriptional response. This experimental setting allows us to catch the most drastic changes at the transcriptional level without significantly killing cells 16,35). From collected samples, mRNAs were extracted, processed, and applied to microarray chips for hybridization 29,35). The statistical analysis revealed 989 genes whose expression was significantly altered, representing approximately 21% of the total ORFs spotted. Compared to the data from the parental wild-type strain under the same condition, only 20 of these 989 genes displayed an opposite transcription pattern (Fig. 2A). In the sequences upstream of genes transcribed at levels significantly different between the wild type and Δ*arcA* strains (> 2-fold), ArcA-binding motifs were identified in general (Table S1). These data suggest that the observed differences at mRNA levels are most likely due to the *arcA* mutation, which overwhelms the impact of H_2_O_2_.

Most members of the OxyR regulon in the *arcA* mutant were still among the most substantially induced, including *katB*, *katG-1*, *ahpC*, *ahpF*, *dps*, *SO1563* and *SO3349* (glutathione peroxidase), *ccpA*, and *ohr* (Table S1) 16. Although *SO1563* and *ohr* are under direct control of OhrR, they are also responsive to H_2_O_2_ 17. These data suggest that ArcA may not co-regulate regulons of OxyR and OhrR. Despite this, as the OxyR-mediated stress response is the most important mechanism for combating H_2_O_2_ in *S. oneidensis*, whether OxyR functions normally in the *arcA*-deficient background needs confirmation. To this end, we compared expression of four genes (*katB*, *ahpC*, *dps*, and *katG-1*) of the OxyR regulon between samples of the Δ*arcA* and Δ*oxyR* strains using a *lacZ*-reporter. While the *ahpC* and *katG-1* genes are positively regulated by OxyR, the *dps* and *katB* genes are under its repression 16. As shown in Fig. 2B, expression levels of all these genes were concertedly upregulated in response to H_2_O_2_ in the *oxyR*^+^ background whereas none of them was responsive in the absence of OxyR. These data conclude that the OxyR regulon is independent of the Arc system in *S. oneidensis*.

### ArcA has little influence on H_2_O_2_ degradation

**Figure 3:**
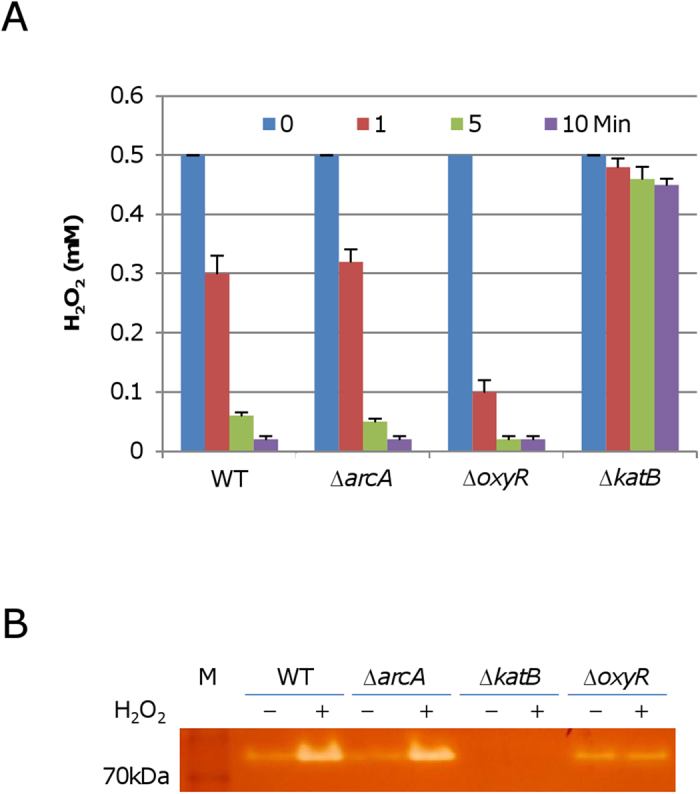
The *arcA* mutation does not compromise the ability to decompose H_2_O_2_. (**A**) H_2_O_2_ consumption assay. H_2_O_2_ at 0.5 mM was added to mid-log-phase cultures, and the remaining H_2_O_2_ at the indicated time points was assayed. The data reported represent the means (*n* *= *4) ± SD. (**B**) Catalase staining analysis. Cells were collected just before and 30 min after the addition of 0.2 mM H_2_O_2_. Proteins from the indicated cell lysates were separated by native PAGE and stained for catalase activity. Experiments were performed three times and similar results were obtained.

In *S. oneidensis*, KatB is the catalase predominantly responsible for degradation of H_2_O_2_ 16. As a heme-containing protein, its activity is subjected to regulation of multiple aspects, such as iron homeostasis, heme biosynthesis, concentrations of endogenous inhibitors. To assess the H_2_O_2_ scavenging capability of the Δ*arcA* strain, we determined H_2_O_2_ consumption of this mutant. Cultures of the Δ*arcA* and its parental wild-type strains at mid-log phase were collected and sonicated. Aliquots of resulting extracts from cells of similar numbers were mixed with 0.5 mM H_2_O_2_ and concentrations of the remaining H_2_O_2_ were measured with time (Fig. 3A). As expected, the Δ*katB* strain lost the ability to degrade H_2_O_2_ almost completely whereas the capacity of the Δ*oxyR* strain was enhanced significantly because of derepression of the *katB* gene 16. In the case of the Δ*arcA* strain, it had a H_2_O_2_-degrading dynamics indistinguishable from that of the wild-type. Moreover, the catalase activities in the wild-type and Δ*arcA* strains were compared. Proteins from cells immediately before and 30 min after the addition of 0.2 mM H_2_O_2_ were separated by native PAGE and stained for catalase activity. Again, the wild-type and Δ*arcA* strains exhibited abilities comparable to each other w/o the H_2_O_2_ treatment whereas the *oxyR* mutant was not responsive to H_2_O_2_ (Fig. 3B). These data manifest that effects of the mutation in the *arcA* gene on the ability of *S. oneidensis* to scavenge H_2_O_2_ is negligible.

### Loss of ArcA results in an increased take-up rate for H_2_O_2_

**Figure 4:**
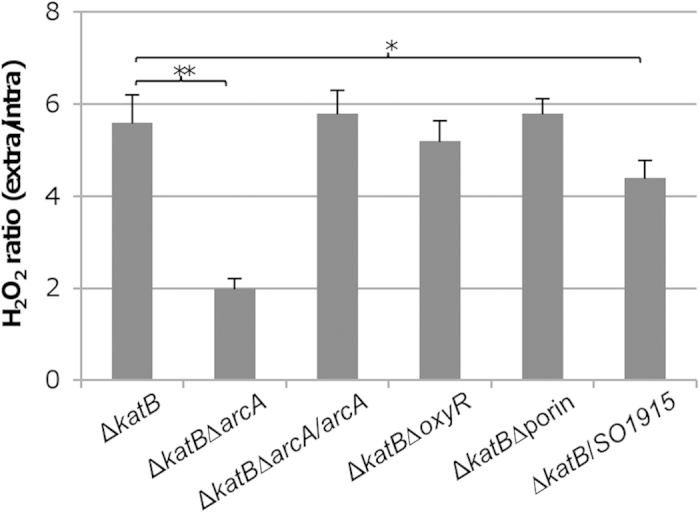
*In vivo* diffusion of H_2_O_2_ into *S. oneidensis*. H_2_O_2_ levels were determined indirectly by specific fluorescence assays in the Δ*katB*, Δ*katB*Δ*arcA*, Δ*katB*Δ*arcA*/*arcA*, Δ*katB*Δ*oxyR*, Δ*katB*Δporin, and Δ*katB*/*SO1915*. Δporin, Δ*arcA*/*arcA*, represent the strains lacking aquaporin as well as all 6 general porins and the genetically complemented strains of Δ*arcA* respectively. Δ*katB*/*SO1915* represents the Δ*katB* strain carrying a copy of SO1915 under control of P*tac* for overexpression with 0.5 mM IPTG. Exponentially growing cells were exposed to H_2_O_2_ (1 mM) for 5 min and peroxide levels were immediately determined in the extracellular (extra) and intracellular (intra) milieu and plotted as the extra/intra ratio. Asterisks indicate statistically significant difference (*P < 0.05; **P  < 0.01; ***P < 0.001). Error bars indicate SD (*n* = 4).

As shown above, neither function of OxyR nor H_2_O_2_ degradation ability in the Δ*arcA* strain is impaired, suggesting that the primary defense system for oxidative stress has no role in the elevated sensitivity to H_2_O_2_ caused by the *arcA* mutation. Given that there exists a H_2_O_2_ gradient across membranes and its diffusion rates have been found to be affected significantly by different membrane compositions 43,44, it is therefore possible that loss of ArcA improves diffusion of H_2_O_2_ across membranes. To test this, a fluorescent scopoletin-based assay was used to quantify H_2_O_2_. Exponentially growing cells of the wild-type and Δ*arcA* strains were treated with 0.5 mM H_2_O_2_ for 5 min and then filtered to separate cells from flow-through to obtain intracellular and extracellular fractions as reported before 42. After quantification of each fraction, the ratio between them was calculated. However, we found that intracellular H_2_O_2_ levels in both strains were too low to be determined confidently. To circumvent this difficulty, we removed the *katB* gene from the ∆*arcA* strain and compared to the *katB* single mutant. The double mutant strain had relatively stable H_2_O_2_ levels in the extracellular portion as the ∆*katB* strain shown in Fig. 3A. This time, significant difference was observed. As shown in Fig. 4, extra/intra ratio of H_2_O_2_ in the *arcA*^+^ strain (Δ*katB*) was 2.8 times higher than in the *arcA*^−^ strain (Δ*arcA*Δ*katB*). In addition, in the same experiment we found that the Δ*oxyR*Δ*katB* strain had a ratio similar to that of the Δ*katB* strain, indicating that OxyR is dispensable in regulating H_2_O_2_ gradient across membranes. These results suggest that deletion of the *arcA* gene facilitates H_2_O_2_ uptake.

It has been reported that the most abundant porin OmpD is found to facilitate transport of H_2_O_2_ across the membrane in *Salmonella enterica* serovar Typhimurium 42. According to a previous report 45, *S. oneidensis* possesses an aquaporin and 6 general porins. To test the role of porins in the uptake of H_2_O_2_, we constructed a strain lacking all of these 7 porins and determined its H_2_O_2_ uptake capacity in the absence of the *katB* gene. As shown in Fig. 4, the extra/intra ratio of H_2_O_2_ of this mutant strain was comparable to that of the wild-type. These results, collectively, indicate that these porins are unlikely to have an important role in the uptake of H_2_O_2_ in *S. oneidensis*, and therefore not responsible for the hypersensitivity resulting from the ArcA loss.

### Loss of ArcA results in a defect in the outer-membrane

**Figure 5:**
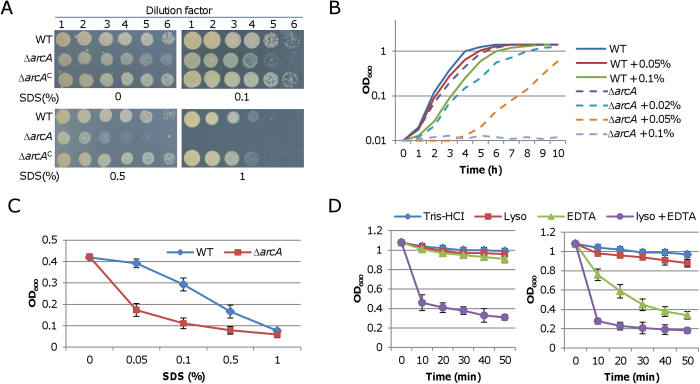
The *arcA* mutation introduces a cell envelope defect. (**A**) Effect of the *arcA* mutation on the SDS resistance in the presence of SDS of indicated concentrations. The assays were repeated at least three times and similar results were obtained. (**B**) Effect of the *arcA* mutation on growth with SDS. Error bars (*n = *4), which were less than 12% of mean in all growth, were omitted for clarity. (**C**) Effect of the *arcA* mutation on the SDS lysis. Cultures of rapid growing (~0.5 of OD_600_) were added with SDS to the final concentrations of 0.05, 0.1, 0.5, and 1%. The OD values were measured 5 min after the addition. The data reported represent the means (*n = *4) ± SD. (**D**) Effect of the *arcA* mutation on the treatment of lysozyme and EDTA. Cells were grown to an OD_600_ of ~1.0, harvested, washed in 50 mM Tris-HCl (pH 7.4), and then treated with either no additions, 0.25 mM EDTA, 100 μg of lysozyme/ml, or both. Absorbance of the cells was monitored at 600 nm. The data reported represent the means (*n = *4) ± SD.

One of possible mechanisms for increased H_2_O_2_ uptake rate is that the cell membranes are impaired. To test this possibility, we first performed sodium dodecyl sulfate (SDS) sensitivity assay. The result demonstrated that the *arcA* deletion drastically elevated susceptibility to SDS (Fig. 5A). In the presence of SDS at 0.1%, the sensitivity of the Δ*arcA* strain to the detergent evidently increased, and at 1% no growth was observed, contrasting a modest augment from the wild-type. Furthermore, the inhibitory effect of SDS on growth of liquid cultures was assessed (Fig. 5B). The loss of ArcA introduced a significant reduction in the aerobic growth rate in rich media, a scenario that is in excellent agreement with the results of previous studies 26,29,30). Compared to those on plates, *S. oneidensis* cells in liquid media appeared much more sensitive to the detergent. No visible growth of the Δ*arcA* strain was observed when 0.1% was served for a 24-h incubation whereas growth of the wild-type was only modestly reduced. The inhibitory effect on the Δ*arcA* strain was evident even with 0.02% SDS. To further confirm that the inhibitory effect of SDS on growth is due to the cell envelope defect, we estimated the lysis of the wild-type and the Δ*arcA* cells by SDS. Cultures of mid-log phase (~0.4 of OD_600_) were treated with SDS of various concentrations and reduction of the optical density was monitored. When SDS was added to 1%, both the wild-type and the Δ*arcA* cultures were lyzed completely (Fig. 5C). In contrast, significant differences in lysis levels were observed when lower concentrations were applied. SDS at 0.05% revealed a most dramatic distinction; the OD_600_ values of the Δ*arcA* culture reduced rapidly whereas the wild-type culture was barely affected.

Although enhanced sensitivity of Gram-negative bacterial cells to SDS is generally attributed to outer-membrane (OM) defects, further evidence is needed to prove whether OM as a permeability barrier is compromised. To confirm the defect in OM, we performed a lysozyme sensitivity assay. In Gram-negative bacteria, the structure of OM is stabilized by interactions between lipopolysaccharide and divalent metal cations; by chelating the latter, EDTA facilitates entry of lysozyme into the periplasm to digest peptidoglycan 46. As shown in Fig. 5D, significant time-dependent lysis of wild-type cells was observed only in the presence of both EDTA and lysozyme whereas EDTA alone was sufficient to induce severe disruption of the mutant cells, let alone rapid and dramatic lysis upon addition of both agents. This observation, in line with the enhanced sensitivity to SDS, indicates that the *arcA* mutation results in a defect in OM.

We then addressed whether the inner-membrane (IM) is defect or not. Microarray data given in Table S1 show a strong induction of the synthesis of PspA upon exposure of the wild-type to H_2_O_2_, which is drastically diminished in the mutant. PspA is induced upon dissipation of the proton motive force (*i.e.* leakage of the inner membrane) and PspA helps restore membrane integrity 47. To evaluate whether the failure of the *arcA* mutant to induce *pspA* expression is the reason for its increased sensitivity to H_2_O_2_, we assessed roles of PspA and/or the proton motive force in SDS and H_2_O_2_ resistance by using an IPTG-inducible expression vector pHGE-P*tac*, which is routinely used in *S. oneidensis* 24,37,48,49). Results showed, as presented in Fig. S1, that PspA produced at varying levels or the loss of the proton motive force did not significantly impact the SDS and H_2_O_2_ sensitivities of either the wild-type or *arcA* mutant strains. We then repeated the sensitivity assay with Triton X-100, a nonionic surfactant that disrupts the inner membrane but not the outer membrane 50, and found that the wild-type and ∆*arcA* strains displayed comparable sensitivities (Fig. S1). These data together conclude that the *arcA* mutation does not interfere with the inner membrane.

### Membrane-bound protease SO1915 in overabundance has a role in the cell envelope defect of the Δ*arcA* strain

The data presented thus far strongly suggest that the increased sensitivity of the Δ*arcA* strain to H_2_O_2_ is likely due to the impaired cell envelope. Given that the phenotype is clearly a result of the *arcA* mutation, we made an attempt to search for ArcA regulon members that may be involved in the cell envelope biosynthesis. Among genes possessing i) a predicted ArcA-binding motif within their upstream sequences and ii) a significant difference in expression (3-fold or above) between the wild-type and mutant strains 16,29), three serine protease genes, *SO0867*, *SO1915*, and *aprE* (SO3106) were particularly intriguing since they all displayed expression differences of over 30-fold (Table S1). Based on the protein subcellular localization prediction by using PSORTb, SO0867 and AprE are extracellular proteins but SO1915 likely resides in OM (data not shown).

**Figure 6:**
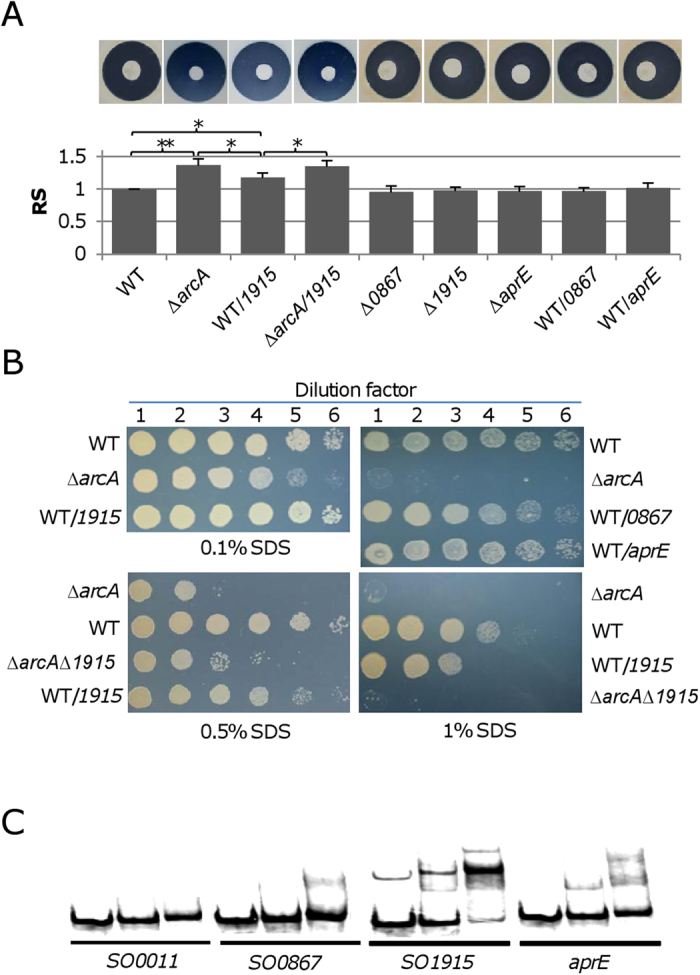
Effects of production changes of three proteases of the ArcA regulon on the cell envelope defect. All assays were repeated at least three times and similar results were obtained. In A and B, strains without overexpressing proteases carried an empty vector. Overproduction was achieved by 0.5 mM IPTG. (**A**) Effects of expression changes of three proteases on H_2_O_2_ inhibition. Experiments were conducted and data were presented as described in Fig. 1. The values are the mean ± S.D. (error bars) (*n *= 4). (**B**) Effects of expression changes of three proteases on the SDS resistance. (**C**) EMSA assay. Experiments were performed in the presence of 0, 1, or 2 μM ArcA-P and 2–5 nM radiolabeled promoter DNA. 0.2 μg/μl poly(dI·dC) was used in all these binding reactions to block non-specific interactions. Promoter region of *so0011* (*gyrB*) was included as negative control. The phosphorylation of the ArcA protein was done with carbamoyl phosphate.

Given that proteases are degradative enzymes which catalyze hydrolysis of target proteins 51, we reasoned that they may be detrimental when in overabundance. To estimate the impacts of these proteases on the oxidative stress response of *S. oneidensis*, we constructed mutants in which each of these genes was in-frame deleted. Neither growth under normal conditions (data not shown) nor susceptibility to H_2_O_2_ or SDS was affected significantly by the loss of each of these enzymes (Figs. 6A,6B). We then tested the ability of these proteins in overproduction to impact the cell envelope with r pHGE-P*tac*. We fused the protease genes to P_*tac*_, resulting in pHGE-0867, pHGE-1915, and pHGE-3106. Each of these constructs was independently transferred by conjugation into the wild-type strain. In the presence of IPTG, cells carrying each of these plasmids were assayed for growth under normal conditions and susceptibility to H_2_O_2_ and SDS (Figs. 6A,6B). With IPTG at 0.5 mM (10~20-fold induction) 24,37,48,49), no difference was observed from strains carrying pHGE-0867 or pHGE-3106. However, overexpression of the *SO1915* gene modestly altered the ability of the wild-type to cope with H_2_O_2_ and SDS although growth was not evidently affected. Interestingly, the H_2_O_2_ and SDS sensitivity of the Δ*arcA* mutant carrying pHGE-1915 was not further increased (Fig. 6A). To confirm this, we examined the H_2_O_2_ uptake of the Δ*katB* strain overexpressing the *SO1915* gene. Expectedly, the extra/intra ratio of H_2_O_2_ was decreased in the presence of 0.5 mM IPTG (Fig. 4). Furthermore, we deleted the *SO1915* gene from the Δ*arcA* mutant and tested susceptibility of the resulting double mutant to H_2_O_2_ and SDS (Fig. 6B). With respective to H_2_O_2_ sensitivity the additional removal did not help significantly, but it did reduce the sensitivity to SDS to some extent, confirming that SO1915 in overabundance has a negative role in the cell envelope integrity. Despite this, it is evident that SO1915 is not the predominant factor accountable for the cell envelope defect resulting from the *arcA* deletion.

Although the presence of ArcA-binding motifs in front of all these three genes suggests that ArcA regulates their expression in a direct manner, a DNA-binding gel shift assay was performed for confirmation as performed before 29. As phosphorylation of ArcA is required for its specific binding, only ArcA (ArcA-P) phosphorylated by carbamoyl phosphate was used. The DNA fragments, approximately 300 bp in length centered by the predicted binding motif of the genes to be tested, were amplified with ^33^P end-labeled primers. It was found that phosphorylated ArcA protein significantly reduced the motility of fragments containing upstream sequences of *SO0867*, *SO1915*, and *aprE* but not the negative control fragment (Fig. 6C). In summary, SO1915 but not SO0867 or AprE in overabundance increases the sensitivity to H_2_O_2_ and SDS although production of all these three proteases is under direct repression of ArcA.

### Cell envelope defect resulting from the *arcA* mutation appears unlikely to rely on a single gene

Obviously, the defect in the cell envelope resulting from the *arcA* deletion is much more severe than that from the overproduction of SO1915 alone, suggesting that the role of ArcA in the cell envelope biosynthesis of *S. oneidensis* is profound and comprehensive. To identify other factors accountable for the cell envelope defect of the Δ*arcA* strain, we intended to introduce a new mutation, aiming at identification of suppressor genes. Plasmid pFAC, a mariner-based transposon vector widely used for construction of random insertion libraries in various bacteria, contains a modest promoter (P_*tn*_) embedded in the transposable sequence 24,52). This additional feature, along with transposon, renders the vector suitability for screening for cryptic or quiescent operons in addition to knockout of active ones. The constructed library was spread on plates supplemented with the proper antibiotics and 0.4% SDS, which allows the wild-type to form colonies in 24 hours and prevents the Δ*arcA* strain from doing so completely. Unfortunately, from a total of ~300,000 individual insertion mutants, estimated by cfu on the SDS-free control plates, we did not obtain a single colony on screening plates. Additionally, we made an attempt with pHGT01, a derivative from pFAC with a robust promoter replacing P_*tn*_ 53. Still, no suppressor strain was obtained. Given that both vectors have been successfully utilized for this purpose by us and other research groups, this result implicates that the envelope defect resulting from the loss of ArcA unlikely relies on a single gene product.

## Discussion

As ROS can be formed intracellularly when molecular oxygen interacts with redox enzymes 54, global regulators for respiration, especially the Arc system, have been implicated in bacterial oxidative stress response. In *E. coli*, *S. enterica* Serovar Enteritidis, and *H. influenzae*, loss of the Arc system has been shown to result in elevated sensitivity to H_2_O_2_ 9,10,42). However, the proposed mechanisms underlying the role played by Arc systems to combat the oxidative stress in these bacteria vary substantially. In *E. coli*, the Arc system is suggested to be important for the resistance to ROS through its pleiotropic effects such as those on metabolism, especially amino acid and/or protein assimilation and synthesis 10. On the contrary, specific effectors are accountable for the increased sensitivity to ROS in *S. enterica* serovar Typhimurium and *H. influenza arcA* mutants 9,11,42). In the former, the most abundant porin, whose expression is negatively regulated by ArcA in the direct manner upon H_2_O_2_ exposure, facilitates uptake of the oxidant. In the latter, the *dps* gene, encoding a well-characterized iron-storage protein of the OxyR regulon, is down-regulated in the absence of the *arcA* gene 1,9,16).

*S. oneidensis* is distinct from *E. coli* in global regulators that are utilized to control respiration. Although Crp is repeatedly shown to play a predominant role and Fnr appears negligible contrasting their *E. coli* counterparts 19,20,21,22,23, both of them have rather limited impacts on the response to oxidative stress. On the contrary, despite the substantial difference between regulons of *S. oneidensis* and *E. coli* Arc systems 29, we showed here that this system is crucial for the bacterium to combat oxidative stress imposed by H_2_O_2_. Like most, if not all, of bacteria, *S. oneidensis* is equipped a large number of the H_2_O_2_ scavenging proteins, all of which are under the control of OxyR and OhrR 16,17. Concerted up-regulation of this entire scavenging repertoire upon H_2_O_2_ exposure in the wild-type and *arcA* mutant strains implicates that the OxyR regulon is functionally independent of the Arc system. Given that the *dps* gene is the under the direct control of OxyR in *S. oneidensis* 16 and is not subjected to regulation by ArcA, it is therefore unlikely that the Arc system of *S. oneidensis* employs the same strategy as *H. influenza* to confer the H_2_O_2_ resistance 9. Additionally, the presented data manifest that porins in *S. oneidensis*, unlike in *S. enterica* serovar Typhimurium, form a major pathway for H_2_O_2_ diffusion across OM 11.

Arc systems are found in γ-proteobacterial species and are now known to be involved in the diverse biological processes 55. Initially, the *arcA* gene of *E. coli* is recognized as the *dye* gene for mutation in this gene confers sensitivity to dyes such as toluidine blue O (TBO) and methylene blue 56. Both TBO and methylene blue are photosensitizers that facilitate ROS generation in the presence of light 57. It has been proposed that the Arc system limits the accumulation of oxygen radicals and the rate of utilization of endogenous reserves 58, which is applied to explain why the *E. coli arcA* mutant is sensitive to redox dyes. However, Alvarez *et al.* 59 have shown that the cellular response to ROS, assuming that it is involved in coping with the stress induced by redox dye, is not sufficient for the enhanced sensitivity. Instead, cytochrome *bd* oxidase, whose expression is positively regulated by the Arc system, is responsible for the resistance of *E. coli* to these redox dyes and reactive nitrogen species (RNS) such as nitric oxide 59,60). Although the *S. oneidensis bd* oxidase appears to function similarly in combating RNS, the *cydAB* operon is under the direct control of Crp rather than ArcA 24,61).

We showed that the cell envelope of *S. oneidensis* is impaired by the loss of the Arc system. The cell envelope defect, based on a substantially increased sensitivity to SDS, leads to an augment of the H_2_O_2_ take-up rate. The enhanced sensitivity of the *S. oneidensis arcA* mutant to H_2_O_2_/SDS is at least in part attributed to over-expressed SO1915, one of three serine proteases under the direct repression of ArcA. In contrast to the other two extracellular enzymes (SO0867 and AprE), SO1915 is located in OM. Conceivably, when overproduced it may exert a detrimental effect on the cell envelope by degrading proteins having a role in maintaining the envelope integrity. Efforts to test this notion by identifying its key targets are underway.

Under our test conditions, overproduction of SO1915 accounts for a small share of resistance of the *arcA* mutant to H_2_O_2_/SDS. We do not yet know the factors for the majority, even with transposon vectors capable of identifying genes of interest by either generating knockouts or overexpressing ones whose transcription is compromised in the absence of ArcA 24,51,52). Therefore, although the predicted *S. oneidensis* ArcA regulon includes a large number of genes encoding proteins in the functional category of cell envelope 20,29,62), our experience argues that none of these can be single-handedly responsible for the observed envelope defect. Nevertheless, we continue our effort to identify the important proteins for the defect as they are the key to better understand the underlying mechanism.

Predicted regulons of *S. oneidensis* (50 operons) and *E. coli* (82 operons) Arc systems differ from each other significantly, sharing only six operons 29,63). We previously proposed that in *S. oneidensis* ArcA-independent expression of conserved genes such as the tricarboxylic acid cycle (TCA) components may be largely due to the loss of ArcA-binding sites in their promoter regions. Similarly, as exemplified by the three proteases SO0867, SO1915, and AprE in this study, *S. oneidensis* ArcA may acquire control over new genes once an ArcA-binding site emerges. Moreover, the sensor protein ArcS (CaChe-PAS-PAS-HisKA) differs substantially from the *E. coli* ArcB (PAS-HisKA) in domain structure. In addition to one extra PAS domain (sensors of diverse signals), a unique CaChe domain may allow the protein to respond to signals other than redox changes 26,27,64). It is tempting to speculate, therefore, that the *S. oneidensis* Arc system senses different external stimuli than its *E. coli* counterpart.

## Author Contributions

H.G. conceived the idea and designed the project. F.W., Y.M., Y.D., L.J. and G.W. carried out the experiments. F.W., G.W. and H.G. analyzed data. F.W., G.W. and H.G. wrote the paper.

## Additional Information

**How to cite this article**: Wan, F. *et al.* Impaired cell envelope resulting from *arcA* mutation largely accounts for enhanced sensitivity to hydrogen peroxide in *Shewanella oneidensis*. *Sci. Rep.* **5**, 10228; doi: 10.1038/srep10228 (2015).

## Supplementary Material

Supplementary Information

## Figures and Tables

**Table 1:** Strains and plasmids used in this study.

**Strain or plasmid**	**Description**	**Reference or source**
*E. coli* strains		
BL21	F^-^ *omp*T *hsd*S_B_(r_B_^-^m_B_^-^) *gal dcm* (DE3)	GE Healthcare
WM3064	Donor strain for conjugation; Δ*dapA*	W. Metcalf, UIUC

*S. oneidensis* strains		
MR-1	Wild-type	ATCC 700550
HG0624	Δ*crp* derived from MR-1	(20)
HG0867	Δ*SO0867* derived from MR-1	This study
HG1070	Δ*katB* derived from MR-1	(16)
HG1328	Δ*oxyR* derived from MR-1	(16)
HG1915	Δ*SO1915* derived from MR-1	This study
HG2356	Δ*fnr* derived from MR-1	(20)
HG3106	Δ*aprE* derived from MR-1	This study
HG3988	Δ*arcA* derived from MR-1	(29)
HG1328-1070	Δ*oxyR*Δ*katB* derived from MR-1	This study
HG1915-1070	Δ*SO1915*Δ*katB* derived from MR-1	This study
HG3988-1070	Δ*arcA*Δ*katB* derived from MR-1	This study
HG3988-1915	Δ*arcA*Δ*SO1915* derived from MR-1	This study
HGPORIN-1070	Δ*aqpZ*Δ*SO1215*Δ*SO1821*Δ*SO3545*Δ*SO3896*Δ*katB* derived from MR-1	This study

Plasmids		
pHGM01	Ap^R^, Gm^R^, suicide vector for mutant construction	(32)
pFAC	Gm^R^, mariner-based transposon vector	(52)
pHG101	Vector for complementation	(33)
pDEST17-ArcA	Expressing vector for *arcA*	(36)
pHGE-Ptac	Inducible expression vector, Km^R^	(49)
pHGEI01	Integrative *lacZ* reporter vector	(37)
pBBR-Cre	Helper vector for antibiotic cassette removal	(24)
pHGT01	Mariner-based transposon vector with an imbedded strong promoter	(53)
pHGE-0867	*SO0867* in pHGE-Ptac	This study
pHGE-1915	*SO1915* in pHGE-Ptac	This study
pHGE-3106	*aprE* in pHGE-Ptac	This study
