# Supplementary material for: Impaired cell envelope resulting from arcA mutation largely accounts for enhanced sensitivity to hydrogen peroxide in Shewanella oneidensis
Source: Sci Rep. 2015 May 15;5:10228. doi: 10.1038/srep10228 (PMC4432559; doi:10.1038/srep10228)
Supplement: Supplementary Information [file srep10228-s1.pdf]

Supplemental materials of

Impaired cell envelope resulting from *arcA* mutation largely accounts for enhanced sensitivity to hydrogen peroxide in *Shewanella oneidensis*

Fen Wan, Yinting Mao, Yangyang Dong, Lili Ju, Genfu Wu, and Haichun Gao[*]

Institute of Microbiology and College of Life Sciences, Zhejiang University, Hangzhou, Zhejiang, 310058, China

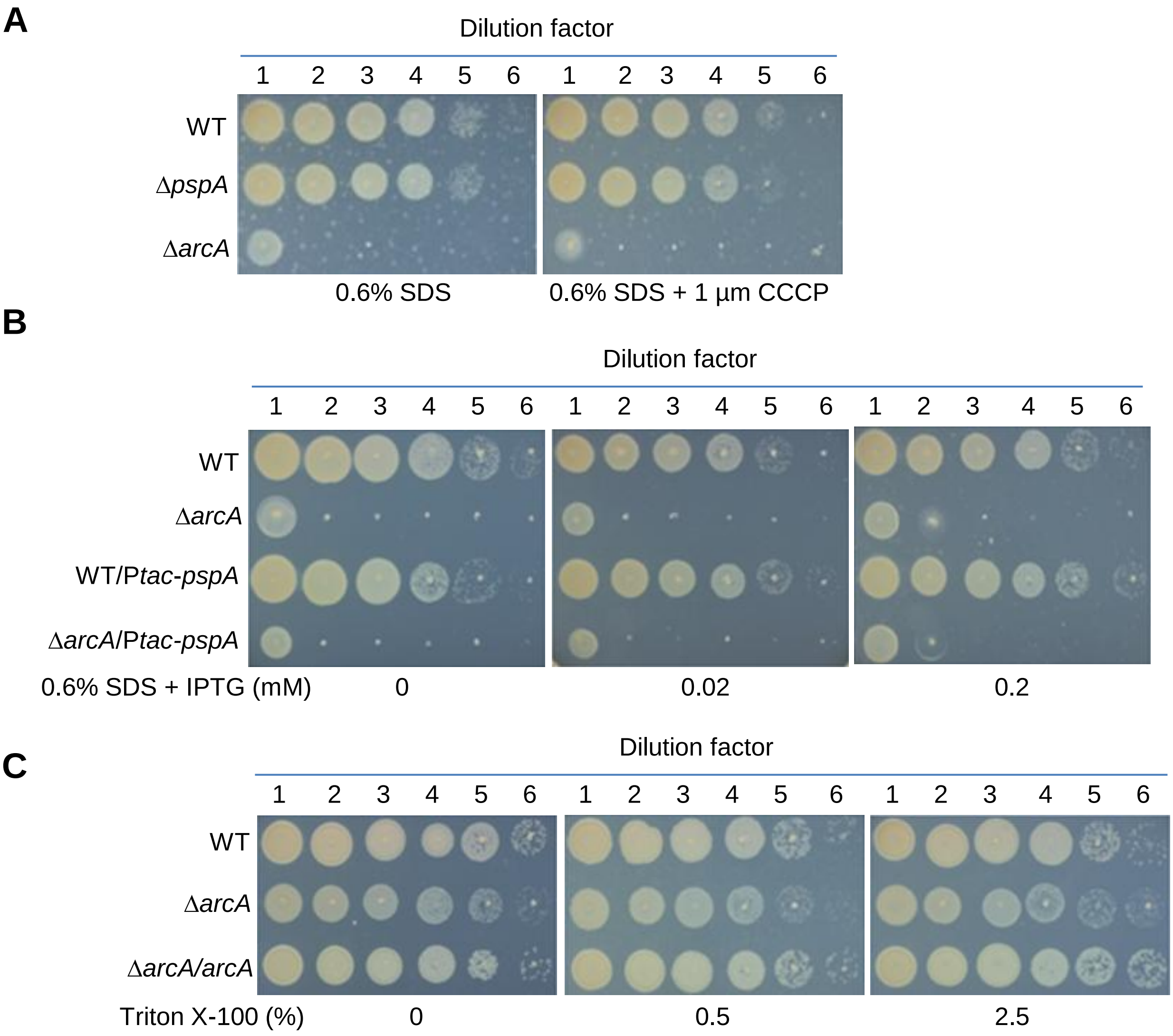

Fig. S1. The *arcA* mutation does not interfere with the inner membrane.

A. Effect of the *pspA* mutation on the SDS resistance with or without carbonyl cyanide *m*-chlorophenylhydrazone (CCCP), an inhibitor of the proton motive force. The assays were repeated at least three times and similar results were obtained.

B. Effect of overexpression of *pspA* on the SDS resistance. The *pspA* gene was under the control of IPTG-inducible P*tac* promoter. According to our calibration, ITPG of 0.2 mM resulted in ~10-fold induction. Error bars (*n* = 4), which were less than 12% of mean in all growth, were omitted for clarity.

C. Effect of the *arcA* mutation on the Triton X-100 resistance. The assays were repeated at least three times and similar results were obtained.

Table S1. Genes that respond to H_2O_2 differently in the wild type (WT) and Δ*arcA* (MT) strains

Locus	Gene	WT	MT	Ratio	FC[a]	Function	Motif[b]	Score[c]
SO0265		0.11	0.55	0.21	T	conserved hypothetical protein	GTTATTAAATTGTGA	10.1
SO0787		1.37	4.93	0.28	T	hypothetical protein	TTTAAAAAGATGTTA	8.6
SO0864		2.37	8.55	0.28	O	transcriptional regulator, LuxR family	GTAATTTAAATGTTA	11.4
SO0866		2.36	90.51	0.03	D	minor curlin subunit CsgB, putative	GTAATTTAAATGTTA	11.4
SO0867		2.78	111.26	0.03	L	serine protease, subtilase family	GTTAAATTACTGTGC	11.4
SO0916		1.36	8.74	0.16	O	transcriptional regulator, MarR family	GTAAATAAAATGTAT	10.1
SO1215		0.76	0.21	3.56	C	outer membrane protein OmpK, putative	GTTAATTATTTGTAT	9.9
SO1415		2.54	0.58	4.36	O	transcriptional regulator, TetR family	GTTAATAAAATGTTT	12.9
SO1807	*pspA*	22.48	3.87	5.80	D	phage shock protein A	GTTAATAAAATGTTT	12.9
SO1812	*mdeA*	7.16	30.56	0.23	H	methionine gamma-lyase	GTAACTAAGTTGTTA	9.6
SO1915		0.93	37.06	0.03	L	serine protease, subtilase family	GTTAAGTAATTGTAA	12
SO2083		0.56	2.51	0.22	D	methyl-accepting chemotaxis protein	GTTAATTAAATGTCA	13
SO2099	*hoxK*	0.03	2.67	0.01	H	Ni/Fe hydrogenase, small subunit precursor	GTTAATTAAATGTCA	13
SO2100		0.35	3.18	0.11	H	thioredoxin family protein	GTTAATAAAAAGTAA	9.1
SO2194		0.01	0.16	0.06	C	OmpA family protein	GTGAATAAAATGTTT	10.7
SO2427		0.56	0.18	3.05	R	TonB-dependent receptor, putative	GTAAACGCTTTGTTA	9.3
SO2445	*thiC*	0.50	0.13	4.02	B	thiamin biosynthesis protein ThiC	GTTATTAAATTTTAA	8.5
SO2628	*cspD*	2.18	18.84	0.12	D	stress response protein CspD	GTTACCTCTTTGTTA	12.8
SO2706	*astB*	0.21	1.14	0.18	H	succinylarginine dihydrolase	GTTGAAAAAATGTAA	9.8
SO3045		0.23	0.77	0.30	T	hypothetical protein	GTTAATTAAATTATA	10.5
SO3106	*aprE*	2.48	127.40	0.02	L	cold-active serine alkaline protease	GTAAATTAATTGTTA	13.1
SO3301		4.18	0.15	27.52	T	flavocytochrome *c* flavin subunit	GTTATTAAGTTGTTT	8.9
SO3480		0.86	3.79	0.23	T	conserved hypothetical protein	GTTAACTCAATGTTA	13.1
SO3561		0.19	1.17	0.16	T	conserved hypothetical protein	GTTAAAACGATGTAA	9.5
SO3562		0.16	0.65	0.24	R	proton/glutamate symporter, putative	GTTAGGCTGTTGTTA	8.6
SO3629		2.86	0.84	3.42	T	expression activator-related protein	GTAAAATCGATGTTA	10.1
SO3694		0.09	0.39	0.22	T	conserved hypothetical protein	GTTGATAAAGTGTAA	9.1
SO3704		0.59	4.38	0.13	T	hypothetical protein	GTTAATTTATCGTTT	9.2
SO3910		16.08	3.48	4.62	T	hypothetical protein	GTTACCACTCTGTTA	9.6
SO4146		1.34	6.84	0.20	L	ABC transporter protein, HlyB family	GTTACCTTTTTGTTT	8.5
SO4457		0.84	3.31	0.25	T	GGDEF domain protein	GTAAATAAAATGTTT	11.3
SO4463		1.10	8.42	0.13	T	prolyl 4-hydroxylase,	GTAAATAAAATGTTT	11.3
SO4512		1.11	0.13	8.47	T	conserved hypothetical protein	GTAAATAAAACGTGA	8.5

[a]B, Biosynthesis of cofactors, prosthetic groups, and carriers; C, Cell envelope; D. Cellular processes; H, Energy metabolism; L, Protein fate; O, Regulatory functions; R, Transport and binding proteins; T, Unknown function.

[b]From (20, 29, 62).

[c]RSAT weight score using Regulatory Sequence Analysis Tools (RSAT) (41).
